# Sperm tRNA-derived fragments: molecular mechanisms and clinical implications for paternal epigenetic inheritance

**DOI:** 10.1186/s13148-026-02155-4

**Published:** 2026-05-14

**Authors:** Shiwei Song, Bin Zhang, Fengli Xiong, Mengkun Li, Lingqi Liu, DongDong Meng, Changfeng Yang, Fulin Ma, Haizhong Xu, Dehui Chang

**Affiliations:** 1 Department of Medicine, Northwest Minzu University, Lanzhou, Gansu, 730050 China; 2 Present Address: Department of Urology, 940th Hospital of the Joint Logistics Support Force, Lanzhou, Gansu, 730050 China; 3 First School of Clinical Medicine, Gansu University of Chinese Medicine, Lanzhou, 730000 China

**Keywords:** tRNA-derived fragments, Paternal epigenetics, Embryonic reprogramming, Transposon silencing, Biomarker, Transgenerational inheritance

## Abstract

Sperm tRNA-derived fragments (tRFs) have emerged as novel regulators of paternal epigenetic inheritance. By modulating embryonic epigenetic reprogramming, transposon silencing, and intergenerational genetic programming, tRFs mediate the transgenerational transmission of paternal environmental information, extending conventional notions of genetic inheritance. This review systematically elaborates the molecular mechanisms, clinical applications, and potential interventional strategies of tRFs. Through an in-depth analysis of how tRFs regulate the epigenetic landscape during embryonic development, we highlight their applications in diagnosing and treating male infertility, as well as in preventing transgenerational diseases. This work provides new insights into interventions targeting paternal epigenetic transmission and associated disorders.

## Introduction

Traditional genetics posits DNA as the sole carrier of genetic information, transmitted to offspring via genes in sperm and oocytes [1–3]. Recent advances in the study of small RNA molecules in sperm have revealed that spermatozoa carry not only DNA but also a novel class of epigenetic regulators—tRNA-derived fragments (tRFs) [4–6]. tRFs are small non-coding RNAs generated through the enzymatic cleavage of mature tRNAs [7, 8] that are abundant in sperm and delivered to the embryo upon fertilization [9]. These fragments play crucial roles in influencing embryonic development and mediating the transmission of paternal epigenetic information. Sharma et al. demonstrated that approximately 80% of small RNAs in mammalian sperm are 28–34 nt in length and are highly enriched in mature spermatozoa [10]. These tRFs respond to paternal environmental changes (e.g., diet, stress, toxin exposure) and enter the oocyte during fertilization to regulate embryonic developmental programs and alter offspring phenotypes [11].

In contrast to traditional genetic perspectives, tRFs serve as vectors of paternal epigenetic information. They mediate the transgenerational transmission of environmental cues by regulating embryonic epigenetic reprogramming, genome activation, and transposon silencing, thereby influencing embryonic development and offspring health [12]. This discovery provides a new perspective on how paternal factors transmitted via sperm can impact developmental and health outcomes in descendants. This review aims to elucidate the critical functions of sperm tRFs in embryonic development, further our understanding of how paternal environment influences offspring development, and provide a theoretical basis for future clinical intervention strategies.

While the discovery of sperm tRFs as epigenetic carriers since 2016 has been pivotal, recent advances have further delineated their specificity and therapeutic potential [10]. This review synthesizes these developments, highlighting the unique role of mitochondrial tRFs (mt-tRFs) in metabolic inheritance and the emerging framework of tRFs as integrators of paternal environmental cues. We incorporate recent insights from models elucidating tRF biogenesis and function [13] to provide an updated perspective on their mechanistic diversity and clinical translation.

## Biogenesis and classification of tRFs

Mitochondrial tRFs (mt-tRFs) mediate the inheritance of metabolic phenotypes.

mt-tRFs act as environmental sensors whose biogenesis is closely linked to mitochondrial metabolic status. Paternal obesity has been shown to significantly elevate levels of sperm mt-tRFs. These elevated mt-tRFs are associated with a reshaped embryonic transcriptome and are proposed to influence offspring metabolic phenotypes [14]. For instance, in high-fat diet (HFD) induced obese mice, sperm mt-tRFs (e.g., mt-tRF-3026b) were increased by 3.8-fold. Through in vitro fertilization, 30% of male offspring developed glucose intolerance. Mechanistically, mt-tRF-3026b directly binds to the 3′UTR of Hk2 (hexokinase 2) mRNA in the embryo, inhibiting its expression and reducing the synthesis of this key glycolytic enzyme. Tomar et al. further confirmed these findings in human cohorts, showing that sperm mt-tRF levels were 2.3 ± 0.4-fold higher (*P* = 0.003) in obese men (BMI > 30) compared to normal-weight controls. Combined with murine IVF experiments, this study provided key evidence that paternal obesity transmits metabolic risk to offspring via sperm mt-tRFs. This process is suggested to alter embryonic metabolic pathways—including glycolysis and fatty acid metabolism—leading to insulin resistance and increased adiposity in offspring [15]. These findings indicate that sperm mt-tRFs can reflect paternal metabolic status and environmental changes, suggesting a potential avenue for mitigating inherited metabolic risk through paternal intervention.

Moreover, mt-tRFs exhibit significant biological functions in specific disease models. For example, in cellular models of MELAS (Mitochondrial Encephalomyopathy, Lactic Acidosis, and Stroke-like episodes), the level of mt-tRF-LeuUUR is closely correlated with mitochondrial respiratory function [16]. Overexpression of this fragment improved mitochondrial respiration, suggesting that mt-tRFs may serve as potential therapeutic targets.

The biogenesis of sperm tRFs involves specific enzymatic cleavage of mature tRNAs by ribonucleases such as Angiogenin (ANG), Dicer, and RNase T2 [7, 8, 17]. Beyond their intracellular generation, a crucial step for their functional repertoire occurs post-testicularly. During epididymal transit, sperm are exposed to epididymosomes – extracellular vesicles secreted by the epididymal epithelium. These vesicles are enriched with diverse small non-coding RNAs, including tRFs, and facilitate their selective delivery and enrichment on or within sperm [9, 18]. This intercellular transfer via epididymosomes is believed to be a key mechanism through which sperm acquire a complex payload of epigenetic information molecules, fine-tuning their regulatory capacity for early embryogenesis.

## Molecular characteristics and functions of tRFs

### Classification and functions of tRFstRFs

tRFs are produced by specific enzymatic cleavage of mature tRNAs and can be categorized as follows (Table 1).

**Table 1:** Classification and functions of tRFs

Type	Generating enzyme	Length	Binding partners	Function(s)	Key references
tRF-3	Dicer	22–24 nt	AGO2	Targets mRNA for degradation or translation inhibition [19, 20]	tRF-3-U
tRF-5	Angiogenin (ANG)	30–33 nt	PIWIL2、MOV10	Silences endogenous retroviruses [17]	tRF-5-GlyGCC
i-tRF	ANG/RNase T2	16–28 nt	RNA-binding proteins	Regulates gene expression and stress response [21]	tRF-3026b (mt-tRF)

### Functional regulation

tRFs play key roles during sperm maturation. The abundance of tRF-5 increases by approximately 300% during sperm transit from the testis to the epididymis, a change closely associated with the acquisition of sperm motility and fertilization potential [18]. Additionally, TET2-mediated m5C modification on tRF-3 enhances its stability and facilitates interactions with effector molecules (e.g., AGO2), ensuring its regulatory functions in the embryo [22]. These coordinated mechanisms ensure the functional specificity and stability of tRFs as epigenetic vectors.

## Molecular mechanisms of paternal epigenetic transmission

### Transcription and translation in early embryos

During early embryonic development, tRNA-derived small RNAs (tsRNAs) play key regulatory roles. Paternal tsRNAs are introduced into the oocyte upon fertilization and participate in zygotic genome activation (ZGA) and early embryogenesis. Some tsRNAs form RNA-induced silencing complexes (RISC) by associating with Argonaute proteins, leading to degradation or translational inhibition of maternal mRNAs and reshaping the embryonic transcriptome [23–26]. Other tsRNAs, particularly those derived from the 5′-end of tRNAs, directly bind to ribosomes and inhibit translation, which may be important under stress conditions. Thus, tsRNAs function not only in post-transcriptional regulation but also in translational control during early embryonic development.

### Role of paternal epigenetic information in embryonic development

tRFs regulate embryonic development through multiple epigenetic pathways, particularly via DNA methylation remodeling and histone modification, ensuring normal development and genomic imprinting.

#### DNA methylation regulates embryonic genes and development

Evidence suggests that tRF-Glu-CTC may bind to the 3'UTR of Dnmt1 mRNA, potentially inhibiting its translation and reducing Dnmt1 expression [27]. Dnmt1 is essential for maintaining DNA methylation patterns during embryonic development. By suppressing Dnmt1, tRF-Glu-CTC reduces DNA methylation levels, promoting demethylation of the H19 gene [26]. H19 demethylation supports normal embryonic development and ensures stable transmission of genetic information [28].

#### Histone modifications regulate gene expression and metabolic pathways

tRFs also influence embryonic development by regulating histone modifications. For example, tRF-Arg-CCG recruits the demethylase KDM3A to promoter regions of metabolic genes, removing H3K9me2 marks and activating sugar metabolism genes [29, 30]. This mechanism helps maintain normal metabolic function and glucose homeostasis in the embryo [31, 32], which is critical for healthy development.

To translate these insights into practical andrology, several concrete strategies can be envisioned. First, tRF profiling could be integrated into routine diagnostic workflows for male infertility. By analyzing the tRF signature in ejaculates, clinicians might identify specific dysregulated pathways (e.g., metabolic, stress-related) contributing to subfertility, enabling more precise subclassification beyond standard semen parameters. Second, for assisted reproductive technologies (ART), targeted tRF modulation presents a novel therapeutic frontier. This could involve the use of liposomes or exosomes as delivery vehicles to supplement sperm with specific ‘healthy’ tRF mimics or to inhibit detrimental tRFs prior to procedures like ICSI or IVF. Such approaches aim to correct the epigenetic information carried by sperm, potentially improving embryo quality and developmental outcomes.

### tRFs in transposon silencing

Transposons are mobile DNA sequences whose uncontrolled activity can cause mutations and genomic instability. Cells employ multiple mechanisms to silence transposons, with tRFs playing a significant role, particularly in sperm. Studies show that tRFs suppress transposon amplification through DNA methylation, histone modifications, and PIWI protein-mediated piRNA pathways [33, 34], thereby ensuring genomic stability.

### Role of tRFs in transgenerational inheritance

**Fig. 1:**
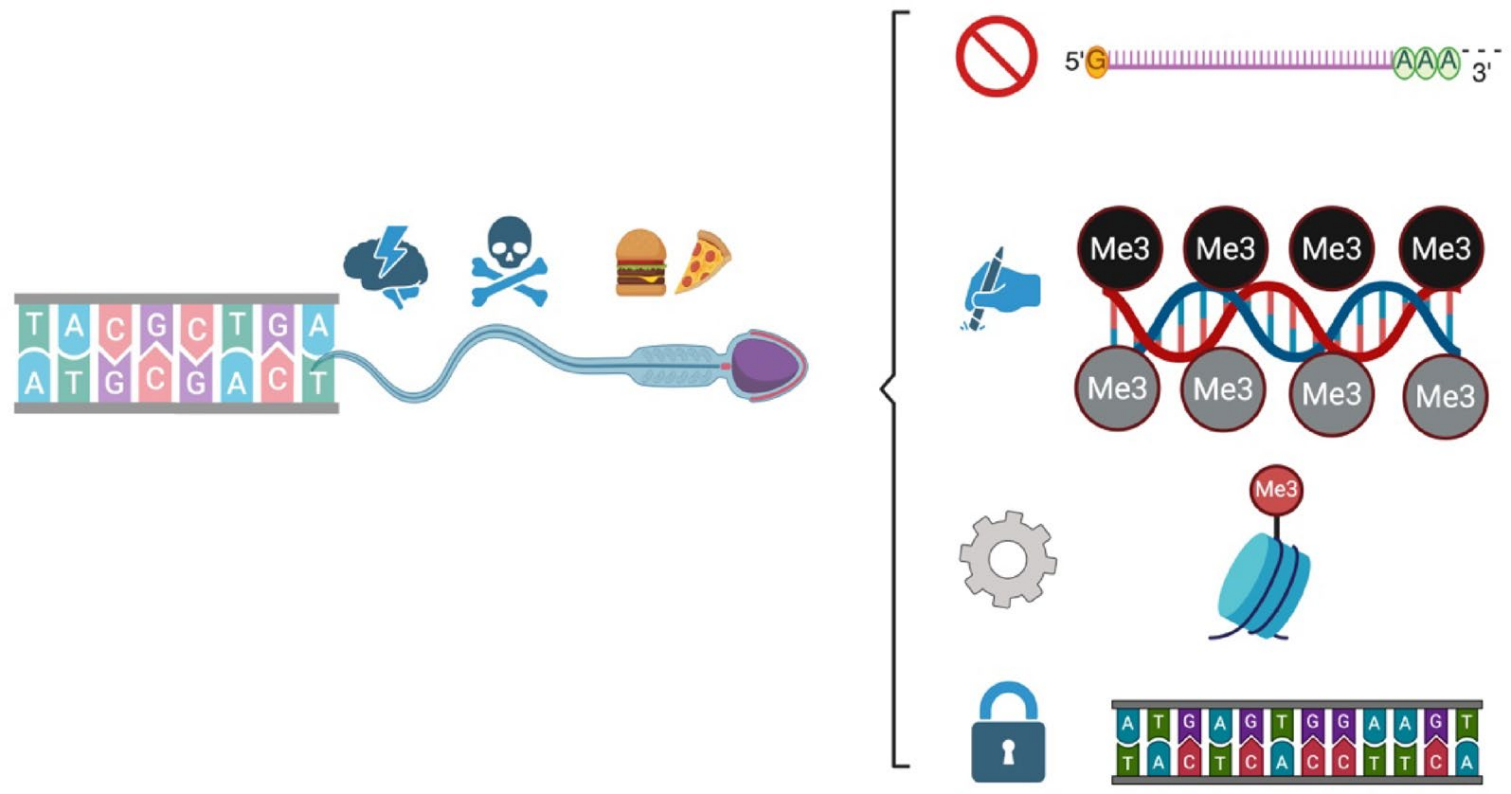
Biogenesis and transfer of sperm tRFs. Schematic illustrating the generation of tRFs from nuclear and mitochondrial tRNAs in sperm, their potential packaging into epididymosomes, and delivery to the oocyte upon fertilization. Created with BioRender.com

Paternal environmental changes (e.g., high-fat diet or stress) modulate tRF expression in sperm, influencing offspring development and health. For instance, paternal high-fat diet elevates sperm tRF-Pro-TGG levels, which impairs offspring pancreatic β-cell function and leads to defective glucose uptake. Paternal stress increases sperm tRF-Leu-CAG levels, which have been associated with altered Bdnf expression in the offspring hippocampus and cognitive deficits, although the precise molecular trajectory from fertilization to postnatal brain remains to be elucidated [35, 36]. These studies demonstrate that paternal environmental factors alter sperm tRFs to affect not only embryonic development but also metabolic and neurodevelopmental outcomes across generations, offering new insights into paternal epigenetic transmission (Fig. 1).

**Fig. 2:**
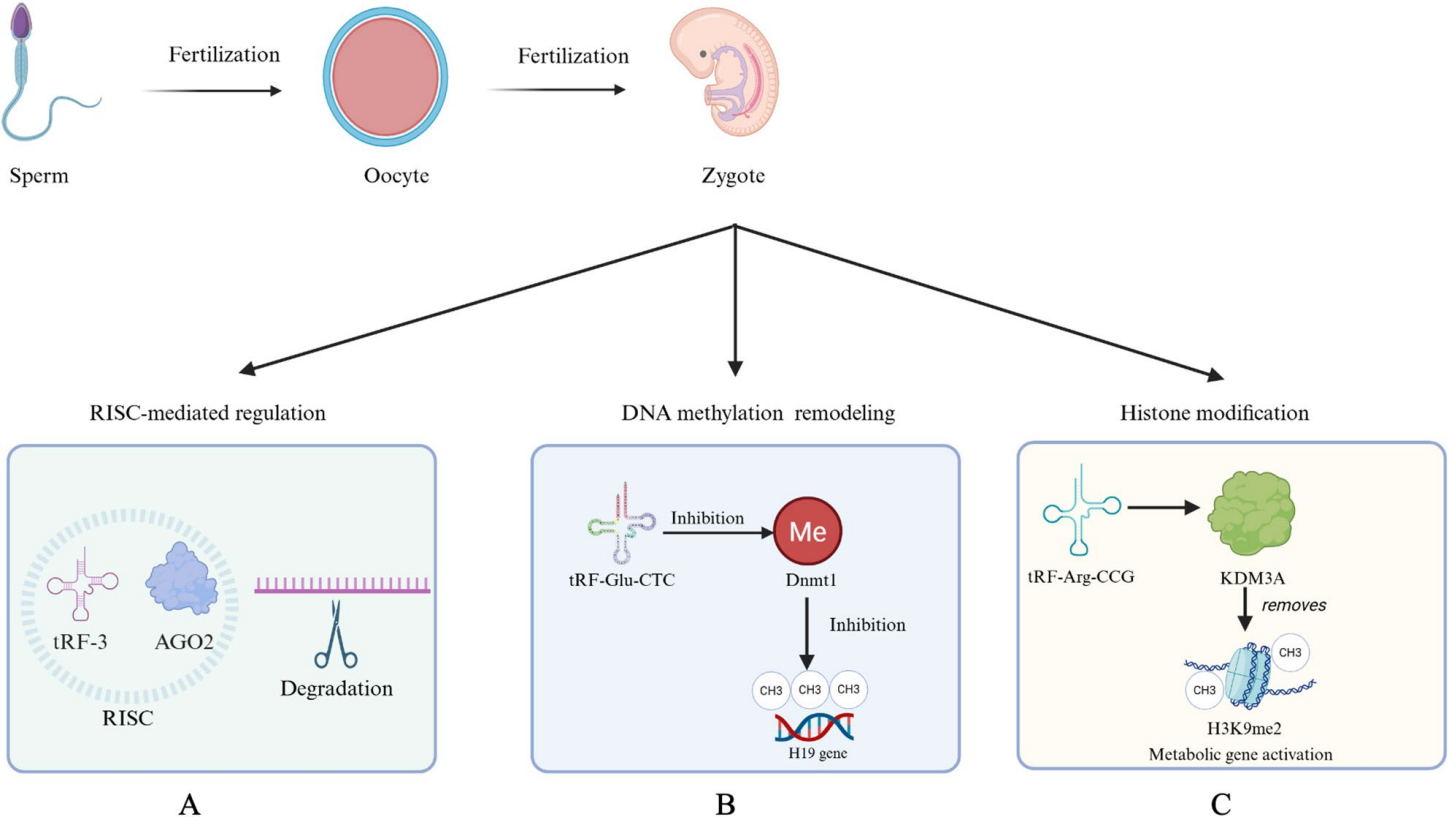
Proposed mechanisms of tRF action in the early embryo. Upon fertilization, paternal sperm deliver tRFs to the oocyte, where they regulate early embryonic development through multiple epigenetic pathways. Based on the findings reviewed in this paper, three major mechanisms are illustrated: **A** RISC-mediated post-transcriptional regulation: tRF-3 fragments associate with Argonaute 2 (AGO2) to degrade or translationally inhibit maternal mRNAs. **B** DNA methylation remodeling: tRF-Glu-CTC suppresses Dnmt1 expression, leading to reduced DNA methylation levels and promoting demethylation of imprinted genes such as H19. **C** Histone modification: tRF-Arg-CCG recruits the demethylase KDM3A to remove H3K9me2 repressive marks from metabolic gene promoters, resulting in gene activation. Dashed arrows indicate hypothesized or indirect pathways

Figure 2 is a schematic of paternal epigenetic inheritance mediated by sperm tRFs.

## Advances in clinical translation

### tRFs as biomarkers for male infertility

Sperm tRFs are closely associated with sperm quality and show promise as diagnostic biomarkers for male infertility. Chen et al. [37] found that tRF-Gln-TTG levels correlate strongly with sperm quality, suggesting its potential as a diagnostic marker. Llavanera et al. [38] highlighted in a systematic review that small non-coding RNAs in semen (e.g., miR-34c-5p) exhibit strong predictive value for diagnosing male infertility. Additionally, certain tRFs (e.g., tRF-Arg-CCG) can improve sperm motility and fertilization capacity by modulating gene expression [39], offering new avenues for diagnosis and treatment. As epigenetic vectors responsive to environmental and lifestyle factors (e.g., diet, stress), tRF expression profiles may serve as novel biomarkers for male infertility and related conditions [40]. These studies highlight the potential of tRFs as novel diagnostic markers for male infertility. Paternal tsRNAs may also play important roles in metabolic diseases (e.g., obesity, diabetes) and neuropsychiatric disorders (e.g., anxiety, autism). Paternal inflammation, aging, or high-fat diet alter sperm tsRNA profiles, which can be transmitted to offspring and affect their health. For example, altered tsRNAs in sperm from aged males led to glucose intolerance, obesity, and anxiety-like behavior in offspring [41–43]. Thus, profiling paternal sperm tsRNAs may serve as a novel biomarker for predicting offspring risk of metabolic or neuropsychiatric disorders, To provide scientific basis for early warning.

While microRNAs (miRNAs) like miR-34c-5p have established predictive value in male infertility diagnostics [38], tRFs offer distinct advantages as biomarkers. Their abundance in sperm, direct response to paternal lifestyle, and role in early embryogenesis position them as complementary or superior indicators for specific infertility subtypes, such as those linked to metabolic or environmental factors.

### Therapeutic intervention strategies

Paternal environmental factors like obesity alter sperm tRF expression and affect offspring metabolic health. Modulating sperm tRF expression can improve the activity of metabolic genes, enhance insulin sensitivity, and improve glucose tolerance. For instance, tRF-Pro-TGG antagonizes the effects of paternal obesity on offspring glucose tolerance [35], improving metabolic health.

tRFs are also implicated in neural development and protection. Sperm tRFs influence offspring neurodevelopment through epigenetic mechanisms. Folic acid supplementation modulates sperm methylation levels and restores BDNF expression [36], improving cognitive function and neuroprotection.

Exosomes as delivery vehicles can efficiently deliver tRF mimics or other small RNAs into embryos, improving blastocyst formation rates and sperm function [44]. This delivery system offers new avenues for restoring sperm function and male fertility.

Detecting tRF expression profiles in sperm or blood allows early identification of infertility risk and enables personalized interventions based on individual tRF profiles.

Beyond regulating gene expression, tRFs may participate in genomic repair. They play roles in transposon silencing and genomic stability. Modulating tRF expression can promote genomic repair, restore sperm function, and improve male fertility [45], providing new directions for sperm DNA repair therapies.

## Research challenges and future directions

Current research on tRFs faces several key challenges, and corresponding solutions and expected outcomes are detailed in Table 2.

**Table 2:** Research challenges and applications of tRFs

Challenge	Solutions	Outcomes
Many tRF target genes and full pathways are unclear [46]	High-throughput single-cell omics combined with CRISPR/Cas9 screening [47]	Mapping tRF molecular mechanisms
Limitations in cross-species translation [48]	Human embryo-like models combined with single-cell multi-omics to simulate human development [49]	Constructing functional maps of tRFs in human embryos
Lack of standardization in sperm tRF extraction, sequencing, and analysis	Establish standardized operating procedures (SOPs)	Improve data quality, comparability, and normalization in tRF research
Ethical considerations for clinical translation	Development of ethical frameworks specific to epigenetic interventions and intergenerational studies; rigorous review board oversight	Responsible translation of tRF-based diagnostics and therapies, ensuring societal trust

Organoid models, nanoparticle delivery systems, and multi-omics databases are future directions for advancing tRF research. Blastoid models can simulate human embryonic development, providing platforms for validating tRF functions [50]. Lipid nanoparticles enhance tRF delivery efficiency and therapeutic efficacy [51]. Establishing transgenerational tracking databases and integrating multi-omics data will elucidate relationships between tRFs and diseases, enabling precision medicine and disease prevention.

Although research on sperm tRF-mediated paternal epigenetics has made ground breaking progress, the field remains in its infancy. Translating basic mechanistic insights into clinical applications represents a major future direction.

Despite the promising findings, several limitations must be acknowledged. Most mechanistic insights are derived from rodent models, and direct evidence in humans remains limited. The causal chain from sperm tRF delivery to specific offspring phenotypes, such as neurobehavioral outcomes, involves multiple intermediate steps that are not fully defined. Additionally, current tRF detection and quantification methods lack standardization across laboratories, which may contribute to variability in reported results. Future studies should prioritize human validation, standardized protocols, and longitudinal tracking of epigenetic reprogramming events.

## Conclusion

Emerging evidence positions tRFs as important epigenetic vectors in sperm, potentially regulating embryonic reprogramming and contributing to transgenerational phenotypic establishment. They are emerging as significant contributors to the molecular dialogue facilitating paternal-offspring information transfer, though their position as a central hub requires further validation. Diagnostic models based on tRFs offer novel tools for precision subtyping of male infertility, while tRF-targeted interventions may open new avenues for preventing and treating transgenerational genetic disorders. Future work should focus on validating tRF-targeting strategies in multicenter randomized controlled trials and deciphering the epigenetic memory mechanisms underlying tRF-mediated transgenerational inheritance. Research on sperm tRFs not only redefines the proactive role of the father in offspring health but also provides a new theoretical framework for understanding gene-environment interactions.

## Data Availability

As this is a review article, no new datasets were generated or analyzed during the current study. Data from referenced studies are available through the respective publications or repositories cited in the references. Not applicable.
